# Adipose Tissue Hyperplasia and Hypertrophy in Common and Syndromic Obesity—The Case of BBS Obesity

**DOI:** 10.3390/nu15153445

**Published:** 2023-08-04

**Authors:** Avital Horwitz, Ruth Birk

**Affiliations:** Department of Nutrition, Faculty of Health Sciences, Ariel University, Ariel 40700, Israel; avitalho@ariel.ac.il

**Keywords:** obesity, Bardet–Biedl syndrome (BBS), hyperplasia, hypertrophy, adipogenesis

## Abstract

Obesity is a metabolic state generated by the expansion of adipose tissue. Adipose tissue expansion depends on the interplay between hyperplasia and hypertrophy, and is mainly regulated by a complex interaction between genetics and excess energy intake. However, the genetic regulation of adipose tissue expansion is yet to be fully understood. Obesity can be divided into common multifactorial/polygenic obesity and monogenic obesity, non-syndromic and syndromic. Several genes related to obesity were found through studies of monogenic non-syndromic obesity models. However, syndromic obesity, characterized by additional features other than obesity, suggesting a more global role of the mutant genes related to the syndrome and, thus, an additional peripheral influence on the development of obesity, were hardly studied to date in this regard. This review summarizes present knowledge regarding the hyperplasia and hypertrophy of adipocytes in common obesity. Additionally, we highlight the scarce research on syndromic obesity as a model for studying adipocyte hyperplasia and hypertrophy, focusing on Bardet–Biedl syndrome (BBS). BBS obesity involves central and peripheral mechanisms, with molecular and mechanistic alternation in adipocyte hyperplasia and hypertrophy. Thus, we argue that using syndromic obesity models, such as BBS, can further advance our knowledge regarding peripheral adipocyte regulation in obesity.

## 1. Introduction

Obesity is a widespread chronic disease classified as a global pandemic. The prevalence of adult overweight and obesity has risen to nearly 40% and 10–15%, respectively, over the past 50 years [1,2]. The development of obesity involves a combination of genetic predisposition and environmental factors, where an individual’s unique genetic background interacts with obesogenic environments [3]. The primary cause of obesity is an imbalance between energy intake and expenditure, disrupting energy homeostasis and resulting in initial fat accumulation in white adipose tissue (WAT) and subsequent accumulation in other tissues. This fat accumulation leads to increased stress and dysfunction in tissues, contributing to the development of obesity-related comorbidities such as type 2 diabetes (T2D), cardiovascular disease, hypertension, liver dysfunction, and cancer, which collectively account for over 70% of premature deaths worldwide [3,4,5,6,7]. The economic burden of obesity includes both direct and indirect costs, including medical expenses, unemployment, and reduced socio-economic productivity [3]. The adipose tissue is distributed throughout the body in two main depots: subcutaneous WAT located beneath the skin, accounting for approximately 90% of total WAT, and visceral WAT located in the abdominal cavity, with the omental depot being the primary visceral fat depot in humans [8]. Central fat distribution around internal organs is associated with a higher risk of metabolic complications and inflammation. The relationship between obesity-related comorbidities and visceral obesity is extensively reviewed elsewhere and falls outside the scope of this review [8,9,10,11,12]. This review focuses on adipose tissue expansion mechanisms in common obesity. Additionally, this review argues for the scientific need to investigate syndromic obesity as a model for the study of adipose expansion, focusing on BBS. Recent findings on BBS, a syndromic obesity, point to the involvement of BBS genes in the hyperplasia and hypertrophy of adipocytes and thus imply a suggestive role of BBS genes in common obesity. Similar to research on monogenic obesity, which resulted in the identification of central genes and mechanisms related to adipose tissue expansion, syndromic obesity should be investigated to advance our knowledge further.

## 2. Adipose tissue Hyperplasia and Hypertrophy

**Scheme 1:**
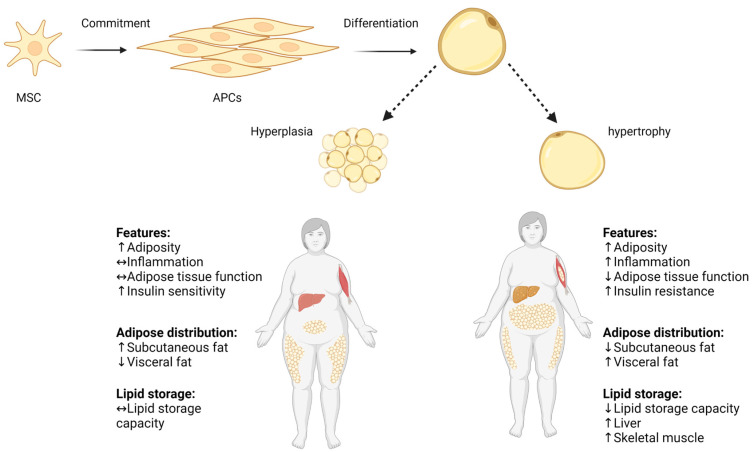
Hyperplasia and hypertrophy in adipose tissue. Pluripotent MSC commit to differentiate into APC lineage located in the stromovascular fraction of adipose tissue, and APCs differentiate into mature adipocyte. Adipose tissue expands by increasing the volume of pre-existing adipocytes (adipose hypertrophy), and by generating new small adipocytes (hyperplasia). Increased adipocyte size correlates with impaired adipose tissue function, increased local inflammation, and decreased lipid storage capacity. ↑ increase, ↓ decrease, ↔ no change. Adapted from “Metabolically Unhealthy Obesity” and “Metabolically Healthy Obesity” by BioRender.com (2022). Retrieved from https://app.biorender.com/biorender-templates (accessed on 16 July 2023).

Obesity is a metabolic state generated by adipose tissue expansion, which can occur through hyperplasia (proliferation and differentiation of adipose precursor cells) and hypertrophy (increase in adipocyte size), as demonstrated in Scheme 1 [13]. The number of adipocytes in a specific fat depot is primarily established early in life and tends to remain stable throughout adulthood [14]. In contrast, adipocyte hypertrophy, caused by a constant surplus of energy, potential is remarkable and can reach a size increase of several hundred micrometers in diameter. Thus, adipose tissue expansion involves a complex interplay of factors including energy balance, genetics, and developmental processes [15]. Adipose tissue expansion can be divided into two main phases: prenatal and postnatal. During the prenatal phase, adipose tissue undergoes dynamic changes characterized by both hyperplasia and hypertrophy processes [16]. Upon gestational week 14, an early fat depot is starting to emerge from connective tissue. The primitive fat depots are composed of primary vessels and proliferating mesenchymal cells that subsequently will differentiate into preadipocytes [17]. Adipocytes begin to appear by the 23rd gestation week, and by the 28th gestation week, discernible fat lobule structures are formed. At the end of pregnancy, fat depots contain and encompass diverse groups of adipocyte subpopulations, primarily distinguished by their fat content and, consequently, their size [18,19]. The postnatal phase begins after birth and continues throughout life, with adipose tissue undergoing dynamic changes in response to energy balance and physiological needs by hyperplasia or hypertrophy [14]. Adipose tissue hyperplasia and hypertrophy depend on and vary with age, with rapid hyperplasia and hypertrophy during early childhood (0–2 years) and adolescence (12–18 years), and with relative hyperplasia stabilization at adulthood, as was shown in longitudinal and cross-sectional studies by Knittle et al. and others [20,21]. The rapid increase in both the hyperplasia and hypertrophy of adipose tissue occurs particularly in subcutaneous depots and is driven mainly by a growth hormone, insulin-like growth factor 1, sex steroids, and nutritional factors [22,23]. Notably, childhood obesity is characterized by accelerated adipose tissue hyperplasia, resulting in the estimated doubling of the adipocyte number compared to normal-weight counterparts, which significantly elevate the risk for the development of obesity in later life as well [23,24]. At normal weight states, the number of adipocytes peaks around puberty, and then stabilizes in adulthood. During adulthood, there is a gradual decline in adipose tissue hyperplasia potential with hypertrophy becoming predominant especially in visceral depots.

Earlier studies suggested that adulthood adipose tissue expansion is only through hypertrophic mechanisms [25,26]. However, recent lineage-tracing models in rodents indicate that under an excess caloric intake, preadipocyte cells can also differentiate into new adipocytes, contributing to adipose tissue expansion via hyperplasia [27,28]. Adulthood hyperplasia activation is possibly driven by the mature hypertrophic adipocytes’ limited oxygen diffusion capacity (which is at most 100 μm), and the neighboring cells’ mechanical stress [29].

Adipose tissue growth, development, and distribution are also sex-dependently mediated by sex hormones such as estrogen and testosterone [30,31]. For example, apart from estradiol fluctuation during the menstrual cycle, impacting appetite, caloric intake, and energy expenditure, it promotes subcutaneous fat accumulation, particularly in the gluteo-femoral region, resulting in the characteristic female pear-shaped body composition [32,33,34,35]. Conversely, testosterone favors the deposition of visceral fat, leading to the typical male apple-shaped body composition [36,37]. Moreover, subcutaneous adipose tissue usually has smaller more ‘plastic’ adipocytes, whereas the visceral adipose tissue is characterized by larger adipocytes encased in fibrotic tissues. Studies of rodents suggest that female mice present more adipose progenitor cells (APCs) in inguinal and gonadal depots compared to males, which respond to diet-induced obesity by elevating APCs’ hyperplasia (probably mediated by estrogen), while visceral fat expands with hypertrophy [8,28,38]. The precise mechanism by which sex hormones determine adipocyte hyperplasia or hypertrophy is still not fully known [30].

Hypoplasia is also a phenomenon related to adipose tissue (although not the focus of the current review), characterized by the underdevelopment or reduced growth of adipose tissue. Hypoplasia results from various factors, including genetic mutations, hormonal imbalances, ageing, or inadequate nutrient availability [39,40]. At the molecular level, adipose tissue hypoplasia can arise from differentiation, proliferation, and survival dysregulation through several key molecular regulators such as peroxisome-proliferator-activated receptor-gamma (PPARγ), CCAAT-Enhancer-Binding Proteins (C/EBPs), insulin-like growth factor 1 (IGF-1), and a fibroblast-growth-factor type (FGF). Additionally, inadequate nutrient availability, particularly essential fatty acids, can also limit adipose tissue growth [41,42,43,44].

## 3. Adipocyte Hyperplasia

Adipose tissue comprises various cell types, including mature adipocytes, stromal cells, fibroblasts, macrophages, blood cells, endothelial cells, smooth muscle cells, mesenchymal stem cells (MSCs), and APCs. APCs, resembling fibroblasts, can differentiate into different preadipocyte lineages (e.g., beige and white adipocytes) in response to genetic and environmental factors, contributing to adipose tissue hyperplasia expansion [45,46,47,48]. The pool of proliferating APCs is located in the stromovascular fraction of adipose tissue and is a depot and sex- and age-dependent [31,40,45,49,50]. Ex vivo and in vivo human studies have identified APC types among CD31− and CD34+ stromal vascular fractions [51,52,53,54,55,56,57]. Similarly, in a mice-specific sub-population characterized by cell surface immune markers was identified to possess proliferation and differentiation pro-adipogenic functional capabilities [51,53,54]. Yet, the final commitment to the adipogenic linkage is not fully known. Adipocytes’ steady-state turnover rate has been under investigation with no consensus to date. In a constant nutritional condition, the suggested estimate is a stable 10% turnover rate yearly [14]. This rate indicates that the maintenance of the adipocyte number normally involves a tightly regulated balance of adipogenesis and adipocyte death [58]. However, shown by several elegant studies, at any life stage, states of over-nutrition lead to adipose tissue hyperplasia and, consequently, to an increased mass [14,50,51,59]. Depot-dependent hyperplasia is a challenging research area due to technical issues; however, generally, it is accepted that a subcutaneous abdominal depot has a higher proliferative and differentiation capacity than a subcutaneous femoral depot and visceral adipose depot [60,61,62]. WAT depot distribution plays a critical role and is a stronger predictor of metabolic health risks than overall obesity. The accumulation of fat in the visceral adipose depot and subcutaneous abdominal depot confers a higher risk of developing T2D and cardiovascular disease, while the accumulation of subcutaneous gluteal and subcutaneous femoral fat may be metabolically protective [59,63,64].

Genetics also plays a role in an individual’s predisposition to adipose tissue hyperplasia, although our understanding on the subject is still evolving, and it is mostly investigated in animal models [65,66,67]. Genetic factors contribute to the development and maintenance of adipose tissue by controlling processes such as cell proliferation, differentiation, and apoptosis. One of the key genetic factors playing a role in adipose tissue hyperplasia regulation is leptin. Leptin-recessive mutated mice (*Ob*/*Ob*) lack leptin, consequently exhibiting hyper-phagic, obesity, hyper-insulinemia, and hyper-glycemia [68]. Furthermore, *Ob*/*Ob* mice present a larger number of adipose cells compared to the control, particularly being pronounced in the female mice [65]. Findings in other genetically obese strains, such as New Zealand (NZO), yellow (*aA^V^*), intermediate yellow (*aA^fY^*), and (*db*/*db*), in states of a high- and low-fat diet show variation in obesity development, suggesting that diet-induced adipose tissue hyperplasia is strain-dependent, and indicating an interaction between genetics and nutrition [69].

## 4. Adipocyte Hyperplasia in Obesity

During life, to replace mature adipocytes and under the condition of an excess energy flow, APC proliferate to generate new adipocytes [70,71]. The proliferation of other stromal cells parallels this process to ensure a sufficient blood flow to supply oxygen and nutrients to the growing tissue [72,73]. The number and size of adipocytes are critical factors in metabolic health. Many smaller adipocytes characterize a better healthy metabolic status, expressed by a better insulin sensitivity, a lower inflammation level, and less ectopic lipid accumulation [74,75,76]. For example, in a prospective study of Pima Indians with either a normal, impaired, or diabetic glucose tolerance, Weyer et al. demonstrated that a smaller adipocyte cell size was positively correlated with an improved glucose tolerance [76]. Enlarged adipocytes are typical of an obese state and correlate with the risk of metabolic syndrome (independent of BMI) [77,78,79]. For example, the overexpression of insulin-responsive glucose transporter (GLUT4) in mice results in obesity and the expansion of body fat only with hyperplasia, accompanied by an improved glucose tolerance [80]. Additionally, the collagen-IV-knock-out (KO) mice, characterized by unrestricted WAT hyperplasia under a positive energy balance due to the weakening of adipocytes’ extracellular scaffold, exhibit a significant weight gain yet an improved insulin sensitivity and inflammatory profile [81]. WAT hyperplasia occurs in a depot-specific manner, as was shown in diet-induced obese mice models [27,82,83]. WAT expansion in an intra-abdominal inguinal fat depot occurs almost exclusively through adipocyte hypertrophy [28], possibly due to the microenvironmental conditions that suppress the APCs’ potential to undergo adipogenesis [84]. In contrast, gonadal WAT can expand in both a hypertrophic and APC hyperplasic manner, triggered by nutrition and specific dietary lipids rather than the total caloric intake [22,82]. Hyperplasia involves complex, sequential, and molecular mechanisms and can be divided into two distinguished processes:

The commitment step—the multipotent precursor mesenchymal stem cells (MSC) differentiate into APCs, which are committed to differentiate into preadipocytes’ lineage. The commitment step is regulated by several signaling pathways and cytokine, as well as epigenetic modifications such as DNA hypomethylation, insulin, glucocorticoids, transforming growth factor β (TGFβ) superfamily members, bone morphogenetic proteins (BMPs), and wingless (WNT) family members [85,86,87]. The commitment stage is a complex process in which gene expression is precisely regulated and is extensively reviewed elsewhere [88,89,90].

The differentiation step—preadipocytes undergo growth arrest, accumulate lipids, and form functional insulin-responsive mature adipocytes [91]. The differentiation transcriptional regulation is a tightly regulated process, accompanied by transit and sequential expression at the level of different transcripts and proteins. This results in the progressive acquisition of morphological and biochemical characteristics of mature adipocytes [92]. Preadipocytes respond to combined mitogenic and adipogenic signals necessary for the subsequent differentiation steps. In the early stage of adipocyte differentiation, the expression of C/EBPβ and C/EBPδ increases, which upregulates C/EBPα expression, further activating PPARγ, the master regulator of adipocytes’ differentiation. PPARγ binds to the retinoic acid X receptor (RXR) to form heterodimers that bind to the PPARγ response element (PPRE) and initiate the transcription of downstream genes, including C/EBPα (positive feedback), to obtain mature adipocytes’ phenotype with the ability to accumulate fat and secret adipokines, such as leptin and adiponectin [93,94].

Adipose tissue hyperplasic expansion is a process regulated by hormones through endocrine, paracrine, autocrine, and neural systems [95,96,97]. Centrally, hormones and cytokines regulate satiety/hunger, metabolic, and activity states in a complex net of interactions. Major central players include glucagon-like peptide-1 (GLP-1), neuropeptide Y, leptin, ghrelin, and Cholecystokinin (CCK) [98]. Peripherally, molecular regulators of the adipose cell number include insulin, PPARγ ligands, retinoids, corticosteroids, and tumor necrosis factor-alpha (TNFα) [92,99,100]. Additionally, adipose tissue is innervated by sympathetic neurons, where APCs’ proliferation is highly responsive to β-adrenergic signaling [101].

States of dysregulation or unbalanced regulation of the complex cascade manifesting adipose tissue development can lead to accelerated adipocyte differentiation and hyperplasia [92,102]. For example, adipocytes are among the most insulin-responsive cell types, thus critically contributing to whole-body insulin sensitivity and energy homeostasis [103,104]. Furthermore, insulin is an obligatory hormone in preadipocyte differentiation to mature adipocytes in both in vivo and in vitro models [105,106]. Obesity induces an insulin resistance state, which is characterized by an adipocyte expansion blockage, possible death, and hyperplasia contributing to obesity co-morbidities, including T2D [107,108]. Growth factors such as insulin-like growth factor 1 (IGF-1) and fibroblast growth factor type 1 (FGF1) are crucial regulators of cell proliferation, survival, and differentiation and participate in the regulation of hyperplasia in adipose tissue [109,110,111]. Alteration in growth hormone levels can lead to the dysregulation of the activation of downstream signaling pathways such as the phosphatidylinositol 3-kinase (PI3K)/AKT and the mitogen-activated protein kinase (MAPK) pathways, which are known to stimulate cell growth and proliferation [112]. Several studies have shown negative correlations between BMI and absolute IGF-1 levels, while others reported increased circulating IGF-1 in obesity and, specifically, abdominal obesity due to elevated portal insulin levels [113,114,115,116,117].

## 5. Adipocyte Hypertrophy in Obesity

Hypertrophic expansion in adipocytes occurs mostly post-developmentally in response to over-nutrition, and is dependent on the ability of existing adipocytes to capture and retain circulating lipids [116]. During calorie restriction, adipocytes provide nutrients to other tissues through the lipolysis of stored lipids and the release of FFAs into the circulation [117]. Adipocyte hypertrophy in obesity is a complex phenomenon expressed not only by the increase in the size of individual cells but also by the remodeling of adipose tissue [48]. Adipocyte hypertrophy leads to dysregulated adipose tissue characterized by a proinflammatory profile and insulin resistance [93,118,119,120]. In the subcutaneous adipose depot, the largest and the least harmful site to store excess calories, expansion is accomplished initially by hyperplasia. However, when hyperplasia capacity reaches its limit, hypertrophy parallels accelerated inflammation and dysfunction. Consequently, excess fat is routed to more detrimental adipose tissue depots, such as visceral depots and ectopic sites [39]. In a “responsive” state, the adipocyte hypertrophy size reaches a plateau that triggers the generation of new adipocytes to accumulate excess fat further and reduce fat “spillover” to other tissues [13,121]. In contrast, states of extreme hypertrophic obesity are characterized by reduced hyperplasia and further hypertrophic expansion, which accelerate obesity co-morbidities [69]. Importantly, the adipocyte size is correlated with metabolic syndrome risk independent of body weight. Thus, hypertrophic adipocytes are characterized by pathologic phenotypes, including hypoxia, fibrosis, the infiltration of proinflammatory macrophages, and chronic inflammation states [78,122,123,124]. Additionally, hypertrophic adipocytes are less responsive to insulin signals, fail to function properly, and produce and secret adipokines [125]. Moreover, the enlarged fat cells in obese adipose tissue have a diminished capacity to store fat. Consequently, the fat “spillover” is directed to other peripheral non-adipose tissues (such as the skeletal muscles, liver, and pancreas), which accumulate the excess “toxic” fatty acids [126,127,128]. Triglyceride (TG) accumulation harms cells and causes cell malfunction due to the activation of inflammatory and stress response pathways (lipotoxicity) and contributes to obesity comorbidity etiology [129,130]. Depot-dependent adipocyte hypertrophy is affected by sex partly through sex-specific hormones, such as estrogen and progesterone [131]. For example, women have smaller adipocytes in omental compared with subcutaneous fat [132], whereas men have a similar size in both depots [133]. Moreover, the maximal adipocyte size was observed in males with a lower BMI compared to women, indicating the preferential storage of excess lipid through adipocyte hypertrophy in men [134]. Under high-fat diet-induced obesity, estrogen supplementation protected only female mice against adipocyte hypertrophy [135]. Adipose tissue expansion mechanisms are illustrated in Scheme 1.

## 6. Molecular Mechanism for Adipocyte Hypertrophy in Obesity

Extensive research, mainly in the mouse cell lines 3T3, has identified critical pathways involved in adipocyte hypertrophy, with the PPARγ signaling pathway standing out as a crucial player [136]. PPARγ is highly expressed in adipocytes and regulates adipogenesis and lipid metabolism genes, such as fatty acid binding protein 4 (FABP4), adiponectin, and lipoprotein lipase (LPL). PPARγ promotes adipocyte hypertrophy by regulating fatty acid storage and suppressing lipolysis through gene regulation, such as HSL and ATGL. PPARγ activity is regulated by fatty acids and signaling pathways known to play a role in adipocyte hypertrophy [137,138,139]. For example, the activation of the extracellular-signal-regulated kinase (ERK) pathway, triggered by growth factors such as insulin and IGF-1, has been demonstrated to regulate PPARγ expression and facilitate adipocyte hypertrophy [140]. In fact, several PPARγ legend drugs, such as thiazolidinediones, have been used clinically to treat T2D, but their long-term use is limited by weight gain and fluid retention [141]. Leptin, an adipokine, is an additional important adipocyte hypertrophy player. However, its role is not fully elucidated, mainly since adipocytes are a significant source of leptin synthesis and secretion and are also prone to leptin resistance. Under a physiological dose, leptin activates the mitogen-activated protein kinase (MAPK) and STAT pathways, promoting adipogenesis and increasing PPARγ2 expression, LPL, and fat storage in rat preadipocytes [142,143]. Conversely, research has shown that high leptin levels inhibit adipocyte proliferation [144]. For example, Wagoner et al. showed that the administration of high leptin levels (250 and 500 ng of leptin/ml) inhibited preadipocyte and stromal vascular cell fraction proliferation in a rat adipose tissue primary culture [145]. However, other studies have also shown that leptin suppresses PPARγ expression in rat adipose tissue [146,147,148].

## 7. Adipose Tissue Hyperplasia and Hypertrophy—Genetic Factors

Genetics is a major factor in obesity etiology; several papers reviewed the subject in detail [149,150,151]. Apart from genetic predisposition to common obesity development, the genetics-estimated contribution to regional fat distribution is 22–61%, indicating a possible role in adipocyte hyperplasia and hypertrophy [152,153,154,155]. It is well established that body fat distribution varies across ethnic groups independent of obesity. For example, in the same obesity category, visceral fat is exhibited more in Asians than Africans and even less in Europeans [153,156,157,158], partly explaining the higher risk of Asians for the development of T2D compared to Caucasians [159]. Furthermore, it might indicate that the subcutaneous fat expansion capacity of Asians is not sufficient to support the growing high-calorie intake [153]. Ethnic Genome-Wide Association Studies (GWAS) on fat distribution have identified genetic predisposition loci that may influence fat distribution and contribute to understanding ethnic-dependent fat distribution diversity (reviewed in [153,155,160,161]). In addition to ethnic genetic background, individual genetic predisposition influences fat distribution regardless of sex and age, as was showed in controlled over-feeding and weight loss studies (refer to [152,162,163,164]). Bouchard et al. [152] reported elegant study findings indicating that heterogeneity in weight gain and fat distribution can vary not only among individuals but also among pairs of monozygotic twins, further highlighting the involvement of genetic factors.

One notable example for individual and ethnic genetic predisposition is the PPARγ gene, which is among the key genes associated with fat distribution. Genetic variations in PPARγ have been linked to metabolic changes and alterations in adipose tissue development. The Pro12Ala variant has been associated with a lower body weight and improved glucose regulation [165,166]. Interestingly, this variant has also been linked to a higher subcutaneous and visceral fat mass in Korean women [167]. In contrast, the Pro115Gln variant has been shown to enhance adipocyte proliferation and triglyceride accumulation in mice, but several epidemiological studies failed to find a clinically significant number of morbidly obese cases bearing this genotype, suggesting that the Pro115Gln variant has a minor effect on morbid obesity development [168,169,170]. Another polymorphism, C478T, within the PPARγ gene, has been associated with higher leptin levels in individuals living with obesity [171], and it has been linked to obesity and an increased fat mass in women [172]. These findings suggest the potential involvement of genetic variations in PPARγ in both the hyperplasia and hypertrophy of adipose tissue.

## 8. The Genetic Contribution to Obesity

Obesity can be divided broadly into two categories:

1. Common polygenic obesity—a common form of obesity, resulting from hundreds of genetic variations in or near many genes, each having a small effect on the risk of obesity development. Polygenic obesity is characterized by late-onset obesity, with a profound environmental influence [173].
2. Genetic obesity—a rare form of obesity, usually inherited in a Mendelian pattern. Genetic obesity is characterized by severe and early-onset obesity, resulting from mutation in a single gene and with small or no environmental influence [174].

**Scheme 2:**
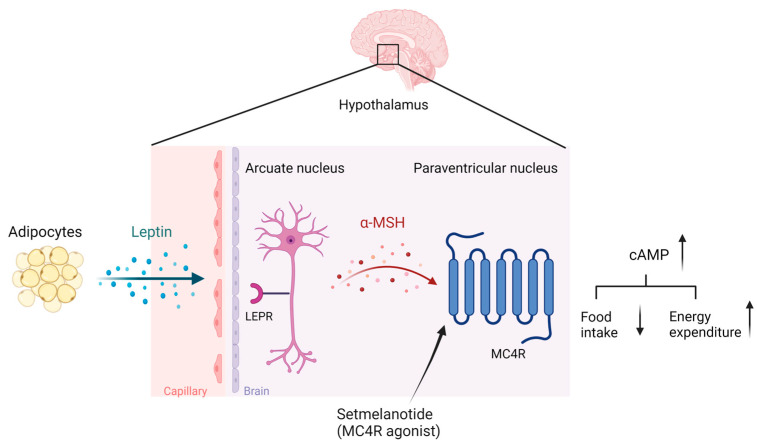
The leptin–melanocortin signaling pathways: Leptin, secreted from adipose tissue, activates leptin receptors on POMC expressing neurons in the arcuate nucleus, leading to the secretion of α-MSH. α-MSH is an agonist of the MC4R expressed in the paraventricular nucleus, up-regulating the conversion of ATP to cAMP and regulating satiety and energy expenditure. Setmelanotide is an α-MSH analog and a pharmacological agonist of MC4R, activating the melanocortin signaling cascade. The scheme was created with BioRender.com.

Even though monogenic obesity is relatively rare, the underlying genetic and biological basis is partly shared with common polygenic obesity [150,175,176,177]. Families, twin, and adoption studies estimated the genetic factor in obesity etiology to be about 40–70% [178,179,180,181]. The common polygenic obesity presents a complex multifactorial pattern with multiple genes’ involvement and gene–gene and gene–environment interactions [182]. Over the last decade, GWASs have identified more than 300 single-nucleotide polymorphisms (SNPs) in or in proximity to genes, partly related to monogenic obesity, that increase or decrease the susceptibility to developing common polygenic obesity [183,184,185,186]. Genetic forms of obesity can be divided further into syndromic or non-syndromic forms. Syndromic obesity is characterized by co-morbidities such as neurodevelopmental disabilities, dysmorphic features, and organ-specific abnormalities [187], while non-syndromic obesity is not accompanied by other specific phenotypes [188]. Non-syndromic obesity is caused by mutations or genetic variants in well-known genes directly regulating energy homeostasis mediated by the leptin–melanocortin signaling pathways in the hypothalamus (Scheme 2) [151]. So far, 16 genes have been identified for monogenic obesity, including leptin (LEP), leptin receptor (LEPR), pro-opiomelanocortin (POMC), prohormone convertase subtilisin/kexin type 1 (PCSK1), melanocortin receptor type 3 (MC3R) and type 4 (MC4R), Melanocortin Receptor Accessory Protein 2 (MRAP2), and adenylate cyclase 3 (ADCY3) [189,190,191,192,193]. Mutations in *MC4R* accounted for 3–5% of all severe human obesity cases [194,195,196]. MC4R is a G-protein-coupled receptor primarily expressed in the central nervous system, including the hypothalamus [197], where it modulates feeding behavior and energy expenditure [198]. Upon MC4R activation by its endogenous ligands, such as α-melanocyte-stimulating hormone (α-MSH), adenylate cyclase synthesizes cyclic adenosine monophosphate (cAMP), resulting in the activation of protein kinase A (PKA) and downstream signaling pathways. These pathways ultimately regulate the expression and function of neuropeptides, such as proopiomelanocortin (POMC), an agouti-related peptide (AgRP), and a cocaine- and amphetamine-regulated transcript (CART), which modulate energy balance and food intake [199]. The leptin–melanocortin signaling pathways are illustrated in Scheme 2. *Mc4r*-KO mice under an ad libitum diet develop hyperphagia and gain significant weight compared to wild-type mice [200]. The re-expression of *Mc4r* in glutamatergic neurons in *Mc4r*-KO mice was sufficient to abolish hyperphagia and decrease body weight [201]. Monogenic non-syndromic obesity accounts for an estimated 5% of severe early-onset obesity [193,202]. Consequently, scientific and pharmacological effort was made to find personalized medication for genetic obesity conditions. In 2020, the Food and Drug Administration (FDA) approved the first treatment involving the MC4R agonist setmelanotide for chronic weight management in adult and pediatric patients at least 6 years of age with obesity due to three rare genetic conditions: *POMC*, *LEPR*, or *PCSK1* deficiency. Setmelanotide is an eight-amino-acid cyclic peptide analog of α-MSH, preferentially binding to and activating *MC4R*, leading to reduced appetite and an increased resting energy expenditure (Scheme 2) [203].

Unlike monogenic mutation, where the mechanisms are mostly known, molecular mechanisms underlying the effect of genetic variations in monogenic obesity genes on the development of common obesity are much less understood [202]. It was suggested that alterations in several pathways are involved, including changes in the levels of appetite-regulating hormones, a decreased leptin sensitivity, a reduced activation of signaling pathways involved in energy expenditure, and an increased food intake [204,205]. GWAS studies found a strong association between common variants in monogenic-obesity-causing genes and the development of common obesity. For example, the SNP rs17782313, located 188 kb downstream of the *MC4R* gene, was found to be significantly associated with a higher BMI, higher fat mass, and elevated risk of common obesity in various populations with different genetic backgrounds, including Caucasians, Mexican, Chinese, and Iranian [206,207,208,209,210,211,212,213,214]. The SNP is suggestively involved in MC4R expression and translation regulation, and consequently affects food intake and nutrient preference [215,216]. However, the exact peripheral effect of monogenic-obesity-related gene variations on adipose tissue hyperplasia and hypertrophy is yet unknown.

A detailed review regarding non-syndromic obesity is beyond the scope of this review and has been previously discussed [151,184,193,217].

## 9. Syndromic Obesity

Syndromic obesity is characterized by a significant heterogeneity and is linked to various molecular mechanisms, such as chromosomal abnormalities (e.g., aneuploidies, microdeletions, duplications, and rearrangements), trinucleotide repeats, imprinting alterations, and monogenic mutations [175]. Over 140 distinct syndromes associated with obesity have been identified, with more than half of them being mapped to specific chromosome regions or locations that involve a causative gene [187,218]. While relatively uncommon, the discovery of genes related to syndromic obesity provides valuable insights for screening and treating common forms of obesity. Furthermore, although not investigated sufficiently, syndromic obesity can shed light on the peripheral mechanisms governing adipose tissue hypertrophy and hyperplasia beyond the central mechanisms. Two well-known syndromes associated with obesity are Prader–Willi syndrome (PWS) and BBS.

**Scheme 3:**
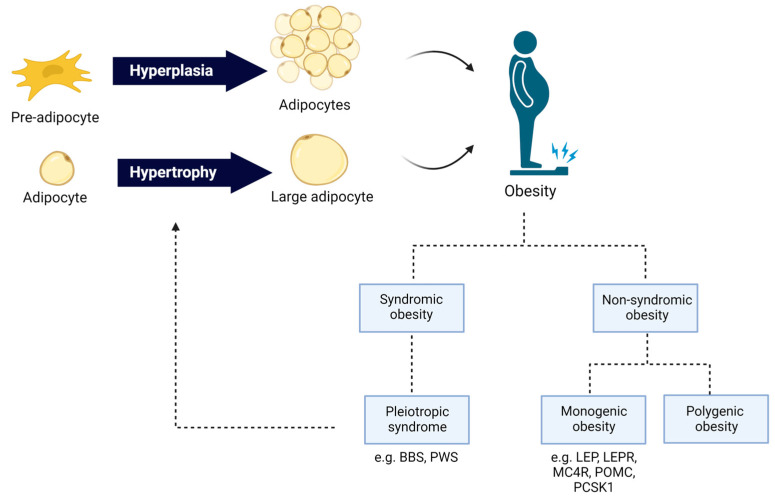
The genetics of obesity. Hyperplasia and hypertrophy of adipose tissue leads to adipose tissue expansion and obesity development. Obesity can be divided broadly into two categories: Common polygenic obesity and genetic obesity. The scheme was created with BioRender.com.

A schematic summary of the genetics of obesity is illustrated in Scheme 3.

Prader–Willi syndrome: PWS is a rare genetic disorder characterized by neonatal hypotonia and an abnormal body composition, with an increased fat mass and reduced muscle mass, endocrine abnormalities (growth hormone deficiency and hypogonadism), intellectual impairment, and learning difficulties, as well as behavioral disorders and dysmorphic traits [219,220]. One of the hallmarks of PWS are eating disorder behaviors, developing in several phases: poor feeding, anorexia with suckling disorders and failure to thrive in early infancy to hyperphagia, and food impulsivity appearing at approximately 4–8 years of age, leading to the development of sever and early obesity [221]. The prevalence of PWS is estimated to be between 1:10,000 and 1:30,000 births, with no gender or ethnic predisposition [222]. PWS is caused by a lack of the gene expression of the paternal chromosome 15q11-q13 region due to several genetic mechanisms. Advanced genetic testing of a large PWS cohort found the deletion of the paternal copy of the 15q11-q13 region in 60% of PWS patients and maternal uniparental disomy (UPD) in approximately 35% of PWS patients, and about 5% is due to imprinting defects or chromosomal rearrangements [223]. Several of the PWS region genes were shown to be involved in the gene regulation of metabolism, growth, and brain function; nevertheless, the functions of the vast majority of the genes remain to be determined [224]. Several mechanisms for the PWS obesity etiology were proposed, including disruption in limbic–hypothalamic pathways of satiety control, resulting in hyperphagia, alterations in hormones regulating food intake, a reduced energy expenditure, and insulin resistance [225]. For example, ghrelin, an orexigenic (appetite stimulant) gut hormone, post-meal plasma levels remain high in PWS patients compared to healthy lean control and non-PWS patients suffering from obesity, specifically prior to obesity onset, leading to a delayed satiety sensation and an uncontrolled drive to eat [226,227,228]. Independent of its orexigenic effects, ghrelin family peptides were shown to induce an accelerated proliferation and differentiation of preadipocytes as well as an increased FFA uptake in adipocytes [229], contributing to adipocyte hypertrophy and hyperplasia as well as lipolysis and lipogenesis in adipose tissue [230,231]. Chao et al. showed that PWS children’s WAT subcutaneous development before the onset of obesity is impaired, with unusual fat expansion, adipocyte hypertrophy, and blunted hyperplasia, due to PWS genetic region noncoding RNA SNORD116 [232]. Similarly, Cadoual et al. found a lower number of WAT APCs in PWS children prior to the development of obesity compared to non-syndromic children with obesity. Although the PWS WAT APC mean number was found to be smaller than that found in non-syndromic children suffering from obesity, WAT adipocytes accumulated significantly more fat after obesity onset compared to non-syndromic children suffering from obesity [233]. Researchers recently found that another deleted PWS loci gene, *Ndh*, negatively regulates the preadipocytes’ ability to differentiate into mature adipocytes, suggestively contributing to increased capacities of APCs for adipogenesis and intensified fat depot hyperplasia in PWS patients [234]. Taken together, initial findings point to the role of PWS region genes in the hyperplasia and hypertrophy of adipose tissue, justifying further research.

Bardet–Biedl syndrome: Bardet–Biedl syndrome (BBS; OMIM 209900) is a rare multisystem, genetically autosomal recessive ciliopathy disorder characterized by obesity, pigmentary retinopathy, polydactyly, renal malformations, mental retardation, and hypo-genitalism [235]. BBS obesity usually begins in early childhood and progresses with age [236]. BBS incidence has an estimated frequency of 1:100,000 to 1:3700 [237,238]. The BBS phenotype represents a challenging genetic basis, since the loss or dysfunction of a single BBS protein can cause a full multi-systemic characteristic of the syndrome. Currently, at least 24 BBS genes have been identified, where the disruption of any of the BBS genes leads to cilia impairment and the disease phenotype [239,240,241]. Most of the known BBS proteins are involved in the formation and function of the primary cilia, a highly evolutionarily conserved organelle functioning primarily for cell-to-cell signaling [242]. Initially, the mechanism regarding BBS obesity was shown to involve a reduced hypothalamic response to leptin, which results in hyperphagia, partly caused by nervous system cilium impairment [243,244]. BBS-related obesity can be partly related to impaired *MC4R* pathway activity; thus, in 2022, the FDA extended the use of setmelanotide for chronic weight management in adult and pediatric patients ≥ 6 years of age with BBS [245]. Recent Phase 3 trial results show BBS patients’ significant decrease in weight and BMI Z score after 1 year of setmelanotide treatment (−7.6% weight change from baseline in patients ≥ 18 years old; −0.75-point BMI Z score change from baseline in patients < 18 years old) [246]. For comparison, POMC- and LEPR-deficiency patients’ mean weight loss after 1 year of setmelanotide treatment was 25.6% and 12.5%, respectively [247]. The relatively lower weight loss of setmelanotide-treated BBS patients compared to setmelanotide-treated patients suffering from mutations in the leptin–melanocortin pathway could be explained by the fact that BBS obesity etiology is not entirely known. Thus, the setmelanotide targeting of the central leptin–melanocortin pathway could suggestively affect the BBS central ciliary dysfunction, a mechanism that failed to entirely explain BBS obesity. Furthermore, BBS proteins have been shown to have additional non-cilium peripheral and central functions that are not related to the leptin–melanocortin pathway [248]. For example, *Bbs2*- and *Bbs4*-null mice present an increased adiposity compared to controls despite pair-feeding adjustments. Furthermore, *Bbs2*-, *Bbs4*-, and *Bbs6*-null mice exhibit high circulating leptin levels at an early age and before the development of obesity [249]. Several studies demonstrated that BBS plays a role in adipocyte biology, including in adipogenesis, indicating that peripheral defects are also involved in the etiology of BBS obesity. Our research has previously shown that *Bbs1*-*Bbs11* transcript levels exhibit a unique and temporal expression pattern during adipogenesis, corresponding to the timing of hormonal induction (insulin and IGF) in the adipocytes in an in vitro model of the 3T3-F442A cell line, highlighting the functional importance of *BBS* genes in adipogenesis [250]. Furthermore, we have shown that *Bbs4* silencing in vitro in preadipocytes induces accelerated cell division and aberrant differentiation and increases aberrant fat accumulation [104,251]. Moreover, we have shown that *Bbs4* plays a role in the ER stress induced in early cell differentiation in vitro in both adipocytes and neuronal cell models [252,253], highlighting that BBS obesity is not only an outcome of a decreased satiety but is mediated in part by peripheral mechanisms, such as accelerated hyperplasia and hypertropia, and the dysfunction or abnormalities of adipocytes.

Even though BBS has a low frequency in the general population, it shares a common phenotype with the obesity epidemic, which is very common in the general population. The heterozygosity of BBS mutations or polymorphisms in BBS genes was shown to contribute to common obesity [186,254,255,256,257,258]. Since obesity development manifested in the intensified hyperplasia and hypertrophy of adipocytes, and aberrant lipid storage, lipogenesis, and lipogenesis processes, it is suggested that BBS obesity is not only related to the central regulation of appetite and satiety mechanisms in the hypothalamus but might be partly due to a direct role of BBS genes in physiological and pathophysiological mechanisms that underlie adipose tissue formation relevant to obesity.

### BBS Effect on Hyperplasia—Reinforcing the Role in Cell Proliferation

The involvement of BBS genes in proliferation and differentiation processes in adipocytes [104,248,251,259,260] is further reinforced when studied in other cell types as well. We have previously reported that BBS genes show a temporal and synchronized expression during adipogenesis, highlighting the importance of BBS genes’ functional role in cell cycle and proliferation processes [250]. We have shown that *Bbs4* plays a direct and essential role in adipocytes’ proliferation. The silencing of *Bbs4* in vitro in mouse preadipocytes (siBBS4 3T3-F442A) induced accelerated cell division, while the over-expression of *Bbs4* in preadipocytes decreased the proliferation rate [251]. These results are in line with our previous findings in the in vitro human neuronal cell line SH-SY5Y (hSH-SY5Y), where BBS4 expression peaks in proliferating undifferentiated SH-SY5Y cells and gradually declines along differentiation. Additionally, siBBS4 hSH-SY5Y cells presented a significant reduction in the expression of early differentiation marker levels compared to levels in control cells [252]. Similarly, we previously reported that BBS4 directly affects adipogenesis, as altered differentiation was observed in *siBBS4* 3T3-F442A adipocytes [251]. Taken together, and in line with other cell types (unpublished data), our consistent findings regarding BBS genes’ role in hyperplasia and hypertrophy in adipocytes, as well as in neural cells, indicate that research on syndromic obesity is essential to identify the novel role of genes involved in central and peripheral obesity.

## 10. Conclusions

Obesity is a chronic multisystem disease, with environmental and genetic factors underlying the basis of its development. The anticipated prevalence and cost of overweight and obesity states will continue to rise in our sedentary, energy-rich environment. Obesity results from adipose tissue hyperplasia and hypertrophy aberration. Common obesity heritability is reported to be 40–60%, corresponding to a relatively high predisposition; however, by nature, it is challenging to study genetic obesity; although rare, it can be used in genetic studies to identify obesity-related genes in order to unravel the complex physiology underlying common polygenic obesity. Monogenetic non-syndromic obesity is often related to the central effect of hypothalamic dysregulation and hyperphagia, leading to uncontrolled eating and, thus, obesity development. In contrast, syndromic obesity is characterized by additional features other than obesity, suggesting a more global role of the mutant genes related to the syndrome and, thus, additional peripheral influence on the development of obesity. BBS is a highly pleiotropic disorder characterized by morbid obesity, and genetic variations in BBS-related genes were shown to contribute to common obesity. BBS genes were shown to influence obesity development in a central effect on leptin signaling and appetite and satiety mechanisms, as well as the peripheral effect on hyperplasia, hypertrophy, and the appropriate function of adipose tissue, leading to the acceleration of adiposeness and augmented fat accumulation. Similarly, and per our previous findings on adipocytes, our current novel results indicate BBS involvement in hyperplasia and differentiation in other cell types. Investigating the global and shared role of BBS genes in hyperplasia and hypertrophy processes in different cell types can partly explain some of the molecular basis of obesity etiology and pathophysiology. We conclude that adopting similar studies involving monogenic syndromic obesity is essential to further understand the role of genes in adipocytes during normal and over-feeding states.

## Data Availability

Not applicable.
